# Region-Based Association Analysis of Human Quantitative Traits in Related Individuals

**DOI:** 10.1371/journal.pone.0065395

**Published:** 2013-06-17

**Authors:** Nadezhda M. Belonogova, Gulnara R. Svishcheva, Cornelia M. van Duijn, Yurii S. Aulchenko, Tatiana I. Axenovich

**Affiliations:** 1 Institute of Cytology and Genetics, Siberian Branch of the Russian Academy of Sciences, Novosibirsk, Russia; 2 Department of Epidemiology, Erasmus MC Rotterdam, Rotterdam, The Netherlands; The University of Chicago, United States of America

## Abstract

Regional-based association analysis instead of individual testing of each SNP was introduced in genome-wide association studies to increase the power of gene mapping, especially for rare genetic variants. For regional association tests, the kernel machine-based regression approach was recently proposed as a more powerful alternative to collapsing-based methods. However, the vast majority of existing algorithms and software for the kernel machine-based regression are applicable only to unrelated samples. In this paper, we present a new method for the kernel machine-based regression association analysis of quantitative traits in samples of related individuals. The method is based on the GRAMMAR+ transformation of phenotypes of related individuals, followed by use of existing kernel machine-based regression software for unrelated samples. We compared the performance of kernel-based association analysis on the material of the Genetic Analysis Workshop 17 family sample and real human data by using our transformation, the original untransformed trait, and environmental residuals. We demonstrated that only the GRAMMAR+ transformation produced type I errors close to the nominal value and that this method had the highest empirical power. The new method can be applied to analysis of related samples by using existing software for kernel-based association analysis developed for unrelated samples.

## Introduction

Genome-wide association studies (GWAS) identified a large number of loci involved in the control of complex traits. However, the results of these studies can explain only a small proportion of trait heritability [1]–[4]. Several new approaches have been proposed to find missing heritability. In particular, the analysis of variants in a region was introduced as an alternative to testing each variant [5], [6]. Simultaneous consideration of a set of single nucleotide polymorphisms (SNPs) from a gene or metabolic pathway addresses the problems of rare variants, computational complexity, and multiple testing, and it simplifies results interpretation and increases the power of the association analysis [7].

Usually, region-based tests use different methods of collapsing rare variants within a region of interest. In this case, a set of rare variants in the region is replaced by a single genetic variable which can then be tested for association with the help of conventional GWAS methods [5], [8]–[10]. The collapsing approach assumes that a large proportion of rare variants is causal and that their effects have the same direction. The power of association analysis decreases if these assumptions do not hold [11].

A new kernel machine regression-based method was recently proposed for conducting regional association analysis [12]–[16]. For quantitative traits, this method compares the average similarity of a set of SNPs from the analyzed region for each pair of individuals with a pairwise phenotypic similarity. The pairwise genetic similarity is measured by using the kernel function which reduces information on multiple SNPs for a pair of individuals into a single scalar factor. Compared with collapsing-based methods, kernel-based methods are more robust to the opposite direction of causal variant effects, a low proportion of causal variants, and the “lower MAF, larger effect size” assumption [16]–[18]. Using family data has long been argued to be of possible benefit in whole genome re-sequencing studies. However, little attention has been paid to the development of kernel-based methods for family data until now. Recently, the method developed for samples of independent individuals [14] was extended to accommodate related samples [19]. The method is based on the variance component approach which was previously proposed for individual SNP-based association analysis [20] as well as for the kernel-based regression for prediction [21] in family data. The distribution of the test statistic under the null hypothesis in this case differs from that in the unrelated samples [19], and existing software for kernel-based regional association analysis could not be used for the related samples. Another approach, which could introduce the data on relatives into kernel-based regression, is a special transformation reducing correlations between phenotypes and between genotypes, suggested by Abney et al. [22] for linear regression analysis of association between individual SNPs and phenotypes. However, a replacement of the real genotypes by the transformed ones disturbs the kernel weight matrix. There is no method that can use the transformed genotypes to build the kernel weight matrix, and therefore, there is no software package implementing kernel-based methods that can test association in samples of related individuals correctly.

Here, we describe a new kernel-based association analysis method for genetically related samples, which is based on a transformation of trait values. This method analyzes related samples by using existing software developed for unrelated samples.

## Materials and Methods

### Model

Let inheritance of a quantitative trait **y** be described by the linear regression mixed model
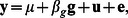
where *μ* is the trait's mean; *β_g_* is the impact of marker genotype **g** on quantitative trait; **u** is polygenic component and **е** is a random environmental effects.

We assume that the quantitative trait follows multivariate normal distribution with vector of means
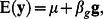
and covariance matrix
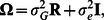
where 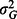 and 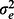 are variance components defined to account for background polygenic and environmental effects, respectively; **R** is a pairwise relationship matrix. Relationship coefficients are defined by a pedigree structure of the sample, or are estimated from the genomic data [23].

In the analysis of the genomic region including a set of *M* SNP markers, the kernel-based score test statistic *Q* is defined as a weighted sum of the individual score statistics 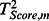 for testing the effects of individual markers on the phenotype under linear regression model [16]:
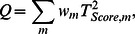
where *w_m_* is a weight of *m^th^* individual marker.

### Score statistic for testing individual SNP's effect

The score statistic for testing association between trait and genotypes of given marker *m* is defined as:
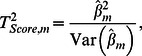
where 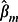 and 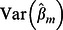 are an effect of marker genotype and its variance in genetically related sample, respectively, estimated as [20]

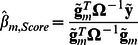
(1)and
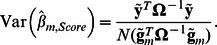
Here *N* is a size of the sample, 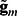 and 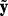 are vectors of centered genotype and phenotype values; genotypes with 0, 1 or 2 minor alleles are coded as 0, 0.5 and 1, correspondently. Thus the score test statistic for genetically related sample is:
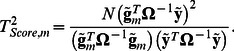
(2)


### GRAMMAR+ transformation

Here we introduce such transformation of phenotype values, 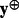, which allows estimating SNP effect and score statistic using simple linear regression:
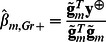
(3)and
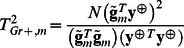
and demonstrate that the estimates (3) approximate the values defined by expressions (1) and (2).

To obtain 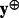 trait transformation, we rewrite expressions (1) and (2) as
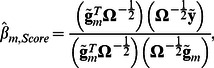
(4)and
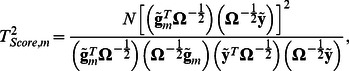
respectively.

The formulas (4) are expressed through two vectors 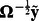 and 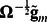. Moreover, we present the vector 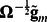 as
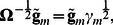
(5)where a *γ_m_* is scalar introduced as
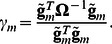
(6)


Since 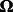 is positive-defined matrix, and 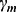 is a positive value, the equation (6) can be rewritten as
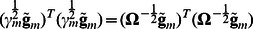
One can see that equation (5) is a solution of last equation.

Recently we demonstrated that when a trait of interest is controlled by a large number (*M*) of the genetic loci of small effect, the values of *γ_m_* for different markers do not differ significantly one from another and may be approximated by averaged value as
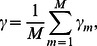
which depends on trait heritability *h* ^2^, total variance *σ* ^2^, and the relationship matrix **R** [24]:
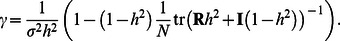
Here we suggest to use the approximation 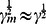 which gives:
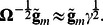
Replacing the vector 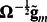 with the vector 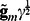 in expressions (4) we obtain
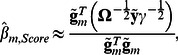
(7)and
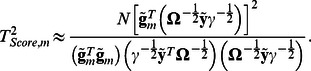



The vector 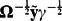 from (7) does not include information about the marker genotypes; it may be calculated once for every analyzed trait. We denote this vector of transformed phenotype values as GRAMMAR+ transformation, 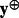. Replacing 
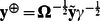
 in formulas (7) gives expressions (3):
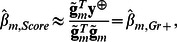
and
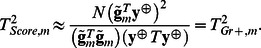



Thus the proposed phenotype transformation allows us to perform the association analysis of genetically related samples using simple linear regression applied in analysis of unrelated samples. GRAMMAR+ transformed traits can be calculated in the ‘polygenic’ procedure in the GenABEL package v 1.7–2 or later (see http://www.genabel.org/ for the GenABEL project web-site).

### Simulated data

To test the new method on exome data, we used the Genetic Analysis Workshop 17 (GAW17 [25]) family sample which consists of 697 individuals in 8 families genotyped for 24,487 exome SNPs in 2,850 gene regions. As 10,703 SNPs were monomorphic in the dataset, only 1,702 gene regions that have more than one polymorphic exome SNP were selected for further analysis. Three quantitative traits (Q1, Q2, and Q4) available from the GAW17 family sample were tested, with Q1 and Q4 being adjusted for modeled covariates prior to the analysis. GAW17 data set includes 200 repeats of simulated traits, which are not enough for estimating the empirical power of different methods. Therefore, we simulated an additional 1000 replicas of Q1 and Q2 by using the GAW17 genotypes of causal loci and models of the trait inheritance described in ref. 25.

Original untransformed traits, GRAMMAR+ trait transformations, and environmental residuals were analyzed by using SKAT R-package [16]. The polygenic model, as implemented in the polygenic function of the GenABEL package, was used to compute the GRAMMAR+ trait transformations and the environmental residuals. The number of polymorphic variants in the GAW17 family data set was not enough to estimate the genomic relationship matrix. Therefore, we used a pedigree structure to estimate within-family kinship. To reduce between-family relationship, we used ten first principal components (PCs) of a pedigree kinship matrix as covariates. The linear weighted kernel was applied with three sets of beta function parameters: (0.5, 0.5), (1, 1), and (1, 25). Test statistics at chromosomes which did not include causal variants were considered as realizations from the null distribution. These empirical null distributions were pooled for all simulations and used to estimate the type I errors and empirical significance thresholds.

### Real human data

We used real data from the Erasmus Rucphen Family (ERF) study, which is embedded in a young genetically isolated Dutch population [26]. The sample included data on 2,596 individuals with a call rate ≥0.95 genotyped on 234,246 autosomal SNP markers with a MAF ≥0.05 and a call rate ≥0.99. We analyzed the following traits: height, body mass index (BMI), serum levels of high-density lipoprotein cholesterol (HDL), low-density lipoprotein cholesterol (LDL), total cholesterol (TC), and triglycerides (TG). All traits were adjusted for age and sex.

SNP markers were analyzed as 23,384 regions obtained with a sliding window of 20 markers shifting by 10 markers. The kernel-based analysis and the trait transformations applied were the same as for the GAW17 data. Genomic kinship was used to run polygenic model and to construct the principal components. The type I error was calculated as a proportion of *P* values not exceeding a given threshold.

## Results

### GAW17 data

**Figure 1:**
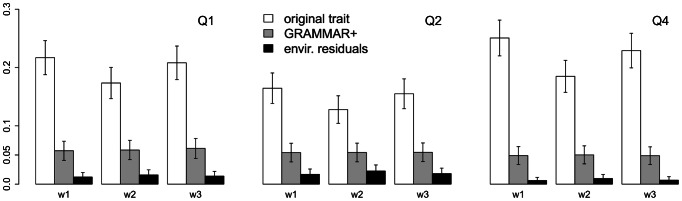
Type I errors for three trait transformations of three GAW17 phenotypes. Different modes of weight function are marked as w1, w2 and w3 corresponding to the parameters of beta function equal to (0.5, 0.5), (1, 1) and (1, 25). Error bars indicate the standard errors.

Type I errors for the analysis of the original traits, GRAMMAR+ trait transformation, and environmental residuals are shown in Figure 1 and Table S1. In GAW17, the Q4 was simulated by using the polygenic model but not specific SNP information and is in that a realization of the null hypothesis. Therefore, Q4 is most suitable for type I error estimation. Under all variants of the weight function, type I errors for the original (untransformed) Q4 were very high. In contrast, the environmental residuals demonstrated conservative type I errors. Only for GRAMMAR+ transformed traits were type I errors for Q4 close to the nominal level for all weight function variants. The same conclusions can be made for Q1 and Q2. However, type I errors for GRAMMAR+ transformations of Q1 and Q2 were slightly higher than the nominal values, which may be explained by the peculiarity of the analyzed sample. In GAW17, many false-positively implicated genes contain variants with exactly the same genotypic distribution as the causal variants used in the simulation model [17]. Such a case is expected when the sample size is much smaller than the number of the rare variants.

When phenotypes were analyzed without PCs as covariates, the type I errors deviated further from the nominal level. The analysis of original traits and GRAMMAR+ trait transformations became more liberal and that of the environmental residuals became even more conservative (Table S1).

**Figure 2:**
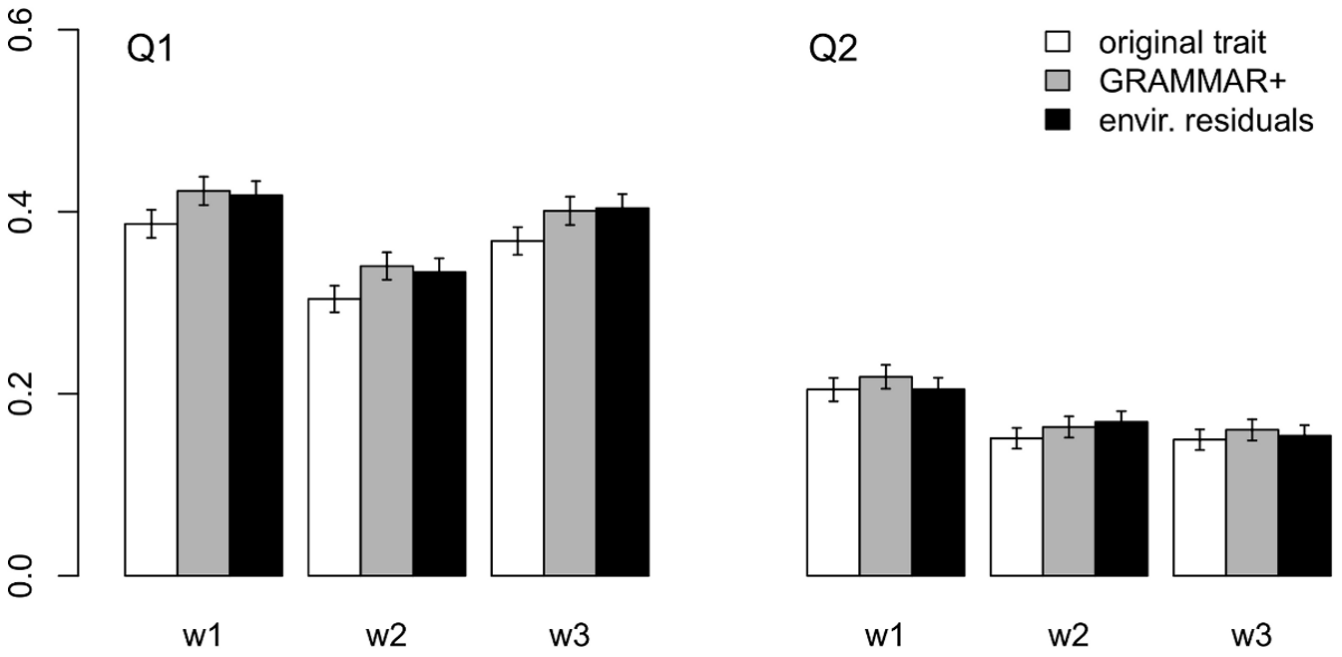
Power for three trait transformations of two GAW17 phenotypes. See legend in Fig. 1 for coding of weight function modes. Error bars indicate the standard errors.

The estimates of empirical power for Q1 and Q2 are shown in Figure 2 and Tables S2 and S3. For all variants of weight function, the empirical power for GRAMMAR+ transformed traits was higher than for the original ones (paired two-sided Student's *t*-test *P* values<0.001 and 0.01 for Q1 and Q2, respectively).

**Figure 3:**
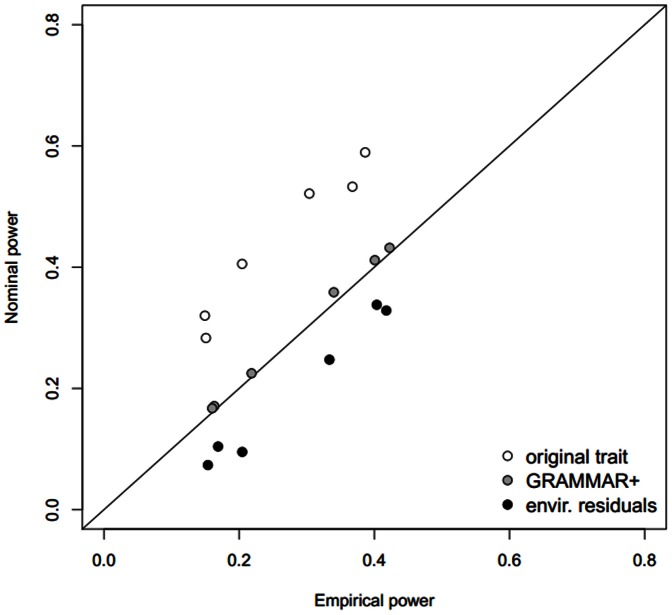
The nominal power plotted against the empirical power for three trait transformations. Each set of six points of the same colour represents the power values for two GAW17 phenotypes (Q1 and Q2) under three different weight function modes. The diagonal line indicates one-to-one correspondence.

We compared the empirical (keeping the empirical type I error fixed at 0.05) and nominal (at α = 0.05, which translates to different type I errors for different transformations) power for the original data, GRAMMAR+ transformations, and environmental residuals (Fig. 3). Nominal power for the original traits was higher than the empirical one whereas that for the environmental residuals was lower than the empirical one. Only for GRAMMAR+ transformed traits were the nominal and empirical powers similar, although empirical power was slightly less than the nominal. As with the slight liberality of the type I errors, this difference between the nominal and empirical powers for GRAMMAR+ transformed data is probably due to the high LD between simulated causal variants and some number of null genetic variants within the GAW17 sample.

In the framework of SKAT software the test's *P* value is estimated by using two approaches, one based on non-central chi-square distribution and other based on bootstrap resampling. We compared *P* values obtained by these two approaches by using the first simulation of Q1 trait and demonstrated that they produce very close estimates for all variants of the trait presentation (Figure S1). The correlation and regression coefficients varied from 0.998 to 0.999 and from 0.996 to 0.997, respectively, for different variants of the traits (transformation).

### Real human data

**Figure 4:**
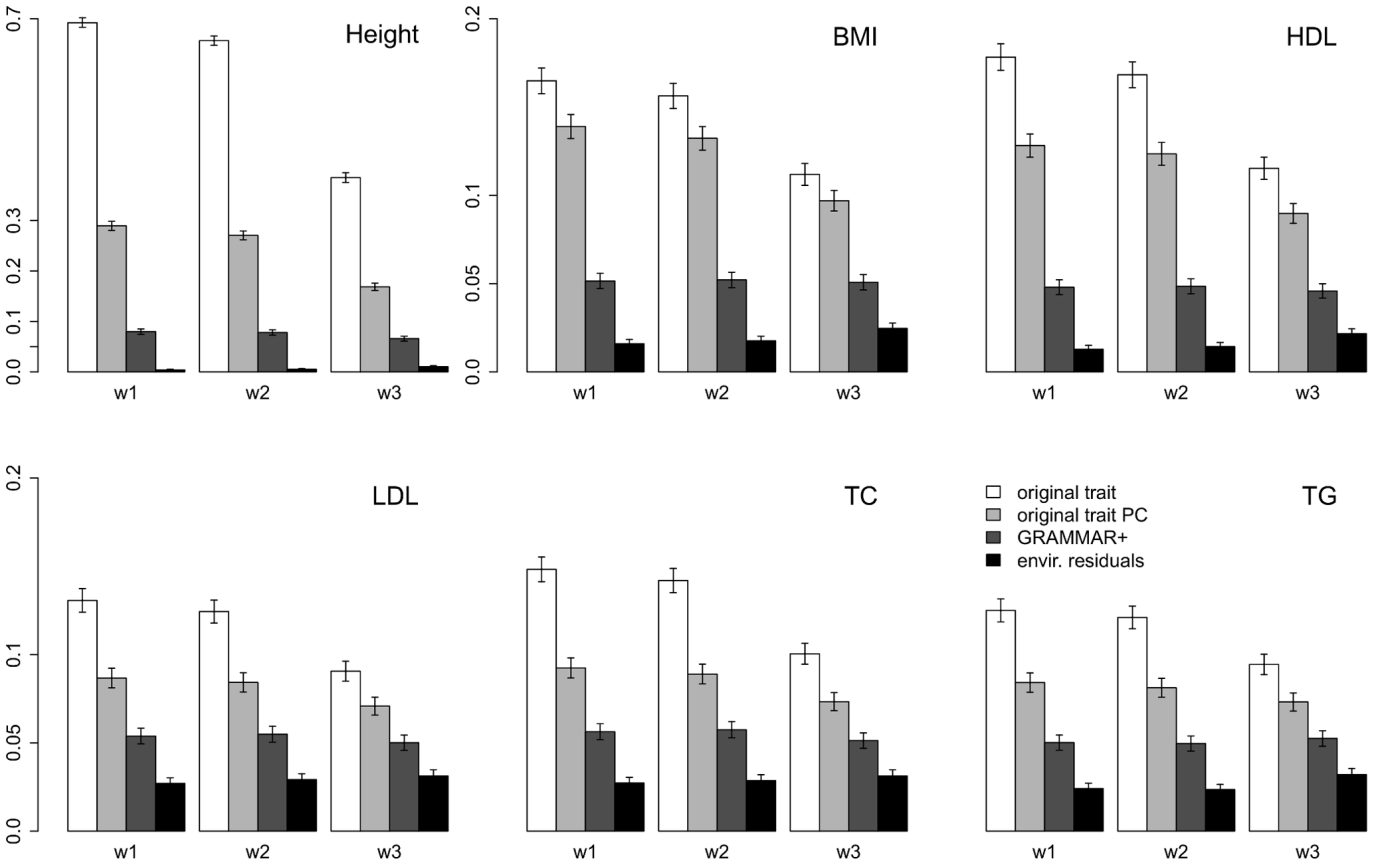
Type I errors for four trait transformations of six human phenotypes. BMI: body mass index; HDL, LDL: high- and low-density lipoprotein cholesterol serum levels; TC: total cholesterol; TG: triglycerides. Different modes of weight function are marked as w1, w2 and w3 corresponding to the parameters of beta function equal to (0.5, 0.5), (1, 1) and (1, 25). Error bars indicate the standard errors.

Figure 4 and Table S4 show proportions of the *P* values≤0.05 obtained for the original, PC adjusted, GRAMMAR+ transformed traits and environmental residuals. The results are very close to those obtained for GAW17 data. Regardless of the variant of the weight function, the proportions of *P* values≤0.05 for the original traits were very high and those for the environmental residuals were very small. When phenotypes were adjusted for PCs, the proportion of *P* values≤0.05 became smaller than for original traits, but still significantly higher than the nominal level. Only for GRAMMAR+ transformed traits the proportions of *P* values≤0.05 were close to the nominal level for all weight function variants. Slightly increased type I error for the GRAMMAR+ transformed height was apparently due to hundreds loci involved in the genetic control of this trait [27].

## Discussion

Our proposed transformation enables the regional association analysis of the data including samples of relatives to be performed by using methods and software developed for analyzing genetically independent samples, e.g., kernel machine-based methods implemented in SKAT. We demonstrated that type I errors obtained for three simulated and six real quantitative traits under three variants of weight function were very close to the nominal values when we analyzed GRAMMAR+ transformed data by using SKAT. Neither original traits nor environmental residuals demonstrated such properties. The theoretical derivation of GRAMMAR+ method was made on the basis of the score test for individual SNP association in samples of relatives; the GRAMMAR+ test can be viewed as an approximation of a more exact GRAMMAR-Gamma method [24]. In the GRAMMAR-Gamma method, gamma factors are assumed to be similar for different markers and may be approximated by their mean. We demonstrated that for the real human traits, this assumption is correct because of the rather small variance of individual gamma factors [24]. Here, we approximate the square root of individual gamma factors by the square root of their mean. Variance for the square root of gamma factors is greater than that for gamma factors because the factors are greater than zero and less than one. Therefore, GRAMMAR-Gamma approximation is the preferred association analysis method for individual SNPs, and GRAMMAR+ transformation expands the range of applicable tools to the case when biased (e.g., because of dependencies in the data) test statistics cannot be simply restored by linear correction.

Regional kernel-based analysis is one of such tools. In this study, we show that the distribution of its test statistic under the null hypothesis does not correspond to the declared distribution when original traits or environmental residuals are analyzed by using methods developed for unrelated samples. Similar behavior was previously found for individual SNP-based association analysis, where ignoring the genetic structure of the sample increases the type I error rate and analyzing independent environmental residuals becomes more conservative [28].

In the framework of single SNP association analysis, the problem of inequality of real and nominal distributions of test statistics under the null hypothesis can be solved in different ways. One of them is based on the genomic control approach. An inflation/deflation factor, which is the ratio between expected values of test statistics for genetically related and unrelated samples under the null hypothesis, does not depend on the allele frequencies under the additive model of trait inheritance [29]. In this case, the inflation/deflation factor can be estimated empirically and easily used for correcting the bias in the test statistic. Distribution of the test statistic for the kernel-based methods under the null hypothesis is rather complex. The distribution is a weighted mixture of chi-square distributions, which can be approximated by non-central chi-square distribution with parameters depending on the analyzed trait, genotypes in the analyzed region, and covariance matrix for related samples [19], [30]. Therefore, in-depth investigations should be conducted to find out whether and how the genomic control method can be introduced into kernel-based analysis of related samples.

Another approach to the problem of inequality of real and nominal distributions of test statistics under the null hypothesis is based on empirical threshold level estimation which can be obtained with the help of resampling techniques. However, special resampling methods which keep the structure of the data intact are needed to guarantee correct empirical estimation of *P* values for the kernel-based methods in case of family data.

The relative sample structure can also be corrected by using a method based on the principal components approach (PCA). This method was proposed to correct the population structure under the kernel-based association analysis [31] because the principal components may be easily introduced into the model as covariates. PCA accurately corrects independent samples from stratified or admixed populations [32], but not for samples with a complex genetic structure [33], such as pedigrees or samples from genetically isolated populations. The results of our investigation support the following conclusion: type I errors remained far from the nominal value when principal components were introduced into a model of inheritance of original traits.

Therefore, none of the known approaches can correct the distribution of the test statistic under the null hypothesis when original traits or environmental residuals are analyzed by using kernel-based methods developed for samples of unrelated individuals. A special method for kernel-based association analysis of related samples was recently proposed by Schifano et al. [19]. To date, this method has not yet been implemented in software. The computational complexity of this method is greater than that of our method because additional multiplication of the covariance matrix is needed to calculate the non-central parameter of chi-square distribution in the case of a related sample. Moreover, non-central parameters have to be estimated for each of thousands of tested regions. Our method multiplies the covariance matrix only once for a given analyzed trait during its preliminary transformation. At the present time, our method is the only method which can be used in practice for kernel-based regional association analysis of samples with related individuals. GRAMMAR+ transformation may be helpful for other association analysis tasks for related samples when software for analyzing related samples does not exist. For example, more complex methods for region-based association analysis, such as optimal tests for rare variants [18] and nonlinear dimension reduction with the Wright—Fisher kernel [34], may be applied to related samples with the help of our method. Moreover, GRAMMAR+ transformation may be a computationally efficient alternative to other methods, including correction on a relative structure in kernel-based analysis via a covariance matrix such as the method by Schifano et al. [19], because our trait transformation allows the use of kernel-based methods with smaller computational complexity.

## Supporting Information

Figure S1 **Nominal versus resampling** ***P*** **value for different trait variants.**
(PDF)

Table S1 **Type I errors for three GAW17 traits analyzed with SKAT.**
(PDF)

Table S2 **Empirical power at causal genes for various trait presentations of Q1 analyzed with SKAT.**
(PDF)

Table S3 **Empirical power at causal genes for various trait presentations of Q2 analyzed with SKAT.**
(PDF)

Table S4 **Type I errors for human height, body mass index (BMI), high- and low-density lipoprotein cholesterol (HDL, LDL), total cholesterol (TC) and triglyceride (TG) levels analyzed with SKAT.**
(PDF)
