# Supplementary material for: Region-Based Association Analysis of Human Quantitative Traits in Related Individuals
Source: PLoS One. 2013 Jun 17;8(6):e65395. doi: 10.1371/journal.pone.0065395 (PMC3684601; doi:10.1371/journal.pone.0065395)
Supplement: Figure S1 — Nominal versus resampling P value for different trait variants. (PDF) [file pone.0065395.s001.pdf]

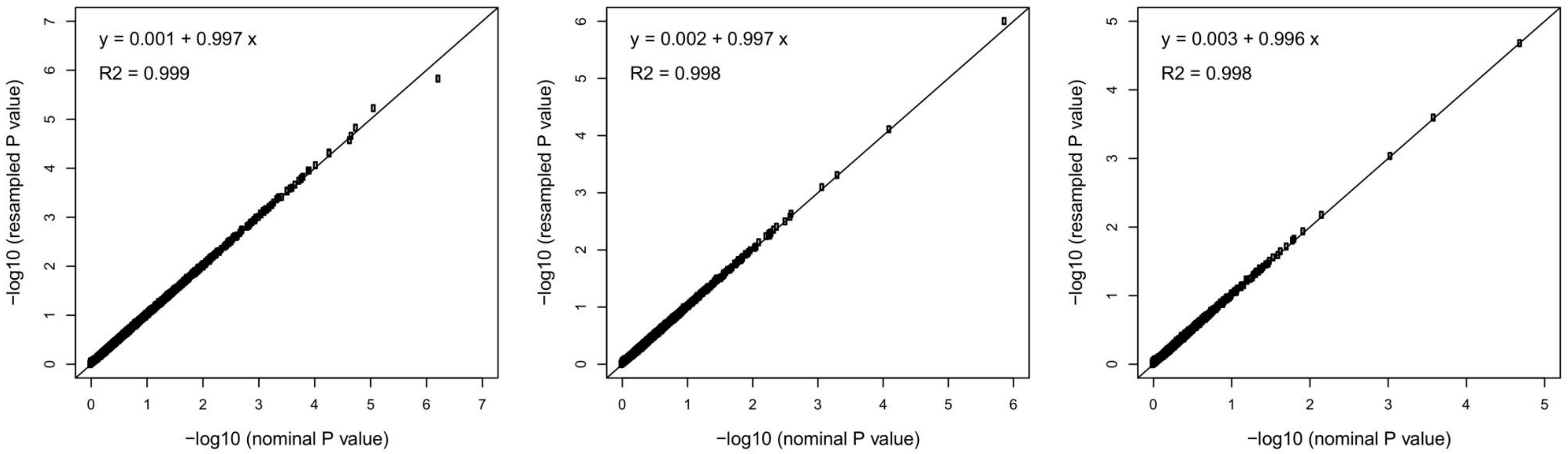

Figure S1. Nominal versus resampling *P* value for different trait variants. Left panel: the original trait; central panel: GRAMMAR+ trait transformation; right panel: environmental residuals. The number of resampling was set as 1000/(*P* value) with upper limit of 10^6.
