# Supplementary material for: Region-Based Association Analysis of Human Quantitative Traits in Related Individuals
Source: PLoS One. 2013 Jun 17;8(6):e65395. doi: 10.1371/journal.pone.0065395 (PMC3684601; doi:10.1371/journal.pone.0065395)
Supplement: Table S1 — Type I errors for three GAW17 traits analyzed with SKAT. (PDF) [file pone.0065395.s002.pdf]

Table S1. Type I errors for three GAW17 traits analyzed with SKAT.

Weights*	Trait presentation	Proportion of *P* values ≤ α					
		α = 0.05			α = 0.01		
		Q1	Q2	Q4	Q1	Q2	Q4
(0.5,0.5)	original trait	0.2168	0.1645	0.2506	0.0952	0.0580	0.1111
	original trait, no PC	0.3803	0.2402	0.3291	0.2402	0.1069	0.1740
	GRAMMAR+	0.0572	0.0542	0.0490	0.0148	0.0114	0.0097
	GRAMMAR+, no PC	0.0857	0.0557	0.0489	0.0287	0.0124	0.0098
	envir. residuals	0.0121	0.0168	0.0062	0.0016	0.0024	0.0005
	envir. residuals, no PC	0.0104	0.0125	0.0045	0.0015	0.0018	0.0003
(1,1)	original trait	0.1734	0.1279	0.1849	0.0672	0.0414	0.0712
	original trait, no PC	0.3405	0.1901	0.2549	0.1946	0.0762	0.1179
	GRAMMAR+	0.0585	0.0543	0.0502	0.0138	0.0115	0.0097
	GRAMMAR+, no PC	0.0828	0.0563	0.0501	0.0240	0.0121	0.0101
	envir. residuals	0.0157	0.0224	0.0097	0.0020	0.0030	0.0008
	envir. residuals, no PC	0.0124	0.0167	0.0067	0.0013	0.0020	0.0005
(1,25)	original trait	0.2080	0.1549	0.2290	0.0908	0.0538	0.0994
	original trait, no PC	0.3255	0.2208	0.3004	0.1985	0.0986	0.1559
	GRAMMAR+	0.0613	0.0546	0.0487	0.0160	0.0115	0.0097
	GRAMMAR+, no PC	0.0889	0.0564	0.0491	0.0329	0.0124	0.0097
	envir. residuals	0.0137	0.0180	0.0068	0.0019	0.0023	0.0006
	envir. residuals, no PC	0.0117	0.0135	0.0049	0.0018	0.0015	0.0004

* Three sets of parameters of beta distribution define three modes of weight function
