# Supplementary material for: Region-Based Association Analysis of Human Quantitative Traits in Related Individuals
Source: PLoS One. 2013 Jun 17;8(6):e65395. doi: 10.1371/journal.pone.0065395 (PMC3684601; doi:10.1371/journal.pone.0065395)
Supplement: Table S2 — Empirical power at causal genes for various trait presentations of Q1 analyzed with SKAT. (PDF) [file pone.0065395.s003.pdf]

Table S2. Empirical power at causal genes for various trait presentations of Q1 analyzed with SKAT.

			Proportion of *P* values ≤ 5% null quantile for causal genes							
Weights*	Trait presentation	5% null quantile**	*ARNT*	*ELAVL4*	*FLT1*	*FLT4*	*HIF3A*	*KDR*	*VEGFA*	all genes (mean)
(0.5,0.5)	original trait	0.002	0.074	0.024	0.975	0.040	0.051	0.568	0.975	0.387
	original trait, no PC	0.000	0.015	0.000	0.792	0.021	0.046	0.617	1.000	0.356
	GRAMMAR+	0.043	0.066	0.040	0.990	0.073	0.041	0.751	0.999	0.423
	GRAMMAR+, no PC	0.026	0.038	0.018	0.984	0.102	0.056	0.836	1.000	0.433
	envir. residuals	0.184	0.054	0.093	0.954	0.058	0.007	0.761	0.999	0.418
	envir. residuals, no PC	0.202	0.045	0.072	0.952	0.087	0.008	0.803	1.000	0.424
(1,1)	original trait	0.004	0.014	0.025	0.135	0.069	0.226	0.667	0.993	0.304
	original trait, no PC	0.000	0.056	0.000	0.093	0.117	0.331	0.716	1.000	0.330
	GRAMMAR+	0.042	0.019	0.028	0.243	0.082	0.206	0.805	0.999	0.340
	GRAMMAR+, no PC	0.029	0.063	0.013	0.208	0.178	0.305	0.857	1.000	0.375
	envir. residuals	0.141	0.038	0.042	0.225	0.064	0.130	0.838	1.000	0.334
	envir. residuals, no PC	0.160	0.046	0.035	0.203	0.132	0.140	0.868	1.000	0.346
(1,25)	original trait	0.002	0.067	0.010	0.993	0.055	0.008	0.448	0.994	0.368
	original trait, no PC	0.000	0.003	0.000	0.875	0.047	0.001	0.172	1.000	0.300
	GRAMMAR+	0.041	0.061	0.015	1.000	0.079	0.010	0.642	1.000	0.401
	GRAMMAR+, no PC	0.024	0.024	0.005	0.996	0.162	0.004	0.578	1.000	0.396
	envir. residuals	0.167	0.048	0.025	0.996	0.076	0.010	0.672	1.000	0.404
	envir. residuals, no PC	0.184	0.049	0.020	0.997	0.146	0.005	0.667	1.000	0.412

* Three sets of parameters of beta distribution define three modes of weight function

** 1174×1000 regions were used to approximate the null distribution
