# Supplementary material for: Region-Based Association Analysis of Human Quantitative Traits in Related Individuals
Source: PLoS One. 2013 Jun 17;8(6):e65395. doi: 10.1371/journal.pone.0065395 (PMC3684601; doi:10.1371/journal.pone.0065395)
Supplement: Table S3 — Empirical power at causal genes for various trait presentations of Q2 analyzed with SKAT. (PDF) [file pone.0065395.s004.pdf]

Table S3. Empirical power at causal genes for various trait presentations of Q2 analyzed with SKAT.

Weights*	Trait presentation	5% null quantile**	Proportion of *P* values ≤ 5% null quantile for causal genes											
			BCHE	*INSIG1*	*LPL*	*PDGFD*	*PLAT*	*SIRT1*	*SREBF1*	*VLDLR*	*VNN1*	*VNN3*	*VWF*	all genes (mean)
(0.5,0.5)	original trait	0.007	0.080	0.016	0.388	0.039	0.036	0.183	0.294	0.219	0.571	0.387	0.038	0.205
	original trait, no PC	0.002	0.077	0.001	0.511	0.039	0.088	0.310	0.209	0.390	0.727	0.284	0.001	0.240
	GRAMMAR+	0.048	0.146	0.033	0.318	0.051	0.029	0.182	0.323	0.220	0.597	0.378	0.128	0.219
	GRAMMAR+, no PC	0.044	0.106	0.029	0.448	0.051	0.045	0.232	0.283	0.319	0.777	0.366	0.085	0.249
	envir. residuals	0.128	0.130	0.055	0.202	0.056	0.018	0.143	0.317	0.197	0.575	0.327	0.233	0.205
	envir. residuals, no PC	0.148	0.106	0.065	0.265	0.049	0.025	0.172	0.261	0.219	0.703	0.297	0.236	0.218
(1,1)	original trait	0.012	0.014	0.034	0.159	0.036	0.023	0.118	0.127	0.041	0.809	0.260	0.041	0.151
	original trait, no PC	0.004	0.013	0.019	0.228	0.032	0.103	0.560	0.072	0.076	0.861	0.115	0.006	0.190
	GRAMMAR+	0.047	0.028	0.029	0.166	0.037	0.038	0.088	0.155	0.043	0.838	0.303	0.073	0.164
	GRAMMAR+, no PC	0.044	0.029	0.024	0.231	0.033	0.093	0.283	0.136	0.049	0.908	0.237	0.042	0.188
	envir. residuals	0.102	0.038	0.029	0.149	0.043	0.055	0.062	0.145	0.045	0.838	0.328	0.128	0.169
	envir. residuals, no PC	0.120	0.042	0.042	0.169	0.028	0.073	0.112	0.164	0.049	0.886	0.284	0.114	0.179
(1,25)	original trait	0.008	0.018	0.008	0.440	0.036	0.027	0.118	0.430	0.231	0.126	0.180	0.031	0.150
	original trait, no PC	0.002	0.055	0.001	0.522	0.031	0.048	0.471	0.352	0.289	0.046	0.112	0.003	0.176
	GRAMMAR+	0.047	0.056	0.028	0.386	0.048	0.028	0.103	0.466	0.252	0.112	0.204	0.081	0.160
	GRAMMAR+, no PC	0.044	0.036	0.025	0.515	0.048	0.046	0.257	0.398	0.290	0.100	0.156	0.042	0.174
	envir. residuals	0.119	0.079	0.053	0.287	0.055	0.027	0.075	0.470	0.221	0.100	0.172	0.155	0.154
	envir. residuals, no PC	0.139	0.053	0.071	0.364	0.051	0.030	0.120	0.379	0.225	0.115	0.133	0.147	0.154

* Three sets of parameters of beta distribution define three modes of weight function

** 641×1000 regions were used to approximate the null distribution
