# Supplementary material for: Region-Based Association Analysis of Human Quantitative Traits in Related Individuals
Source: PLoS One. 2013 Jun 17;8(6):e65395. doi: 10.1371/journal.pone.0065395 (PMC3684601; doi:10.1371/journal.pone.0065395)
Supplement: Table S4 — Type I errors for human height, body mass index (BMI), high- and low-density lipoprotein cholesterol (HDL, LDL), total cholesterol (TC) and triglyceride (TG) levels analyzed with SKAT. (PDF) [file pone.0065395.s005.pdf]

Table S4. Type I errors for human height, body mass index (BMI), high- and low-density lipoprotein cholesterol (HDL, LDL), total cholesterol (TC) and triglyceride (TG) levels analyzed with SKAT.

Weights*	Trait presentation	Proportion of *P* values ≤ α											
		α = 0.05						α = 0.01					
		height	BMI	HDL	LDL	TC	TG	height	BMI	HDL	LDL	TC	TG
(0.5,0.5)	original trait	0.6921	0.1648	0.1783	0.1307	0.1482	0.1250	0.4750	0.0532	0.0584	0.0373	0.0461	0.0366
	original trait PC	0.2895	0.1390	0.1282	0.0867	0.0924	0.0841	0.1229	0.0405	0.0369	0.0216	0.0228	0.0198
	GRAMMAR+	0.0799	0.0515	0.0479	0.0538	0.0563	0.0501	0.0193	0.0097	0.0092	0.0120	0.0128	0.0107
	envir. residuals	0.0040	0.0159	0.0128	0.0270	0.0273	0.0241	0.0003	0.0021	0.0017	0.0051	0.0046	0.0043
(1,1)	original trait	0.6566	0.1563	0.1682	0.1243	0.1419	0.1210	0.4323	0.0497	0.0553	0.0357	0.0440	0.0337
	original trait PC	0.2705	0.1323	0.1235	0.0842	0.0890	0.0812	0.1130	0.0382	0.0349	0.0209	0.0221	0.0183
	GRAMMAR+	0.0780	0.0521	0.0485	0.0549	0.0574	0.0496	0.0191	0.0100	0.0088	0.0118	0.0130	0.0103
	envir. residuals	0.0053	0.0176	0.0143	0.0292	0.0287	0.0235	0.0003	0.0025	0.0015	0.0049	0.0047	0.0041
(1,25)	original trait	0.3851	0.1119	0.1153	0.0906	0.1004	0.0944	0.2132	0.0341	0.0345	0.0245	0.0283	0.0266
	original trait PC	0.1685	0.0970	0.0897	0.0708	0.0733	0.0731	0.0625	0.0287	0.0242	0.0159	0.0166	0.0188
	GRAMMAR+	0.0659	0.0508	0.0458	0.0501	0.0513	0.0525	0.0155	0.0104	0.0100	0.0103	0.0106	0.0106
	envir. residuals	0.0103	0.0246	0.0216	0.0313	0.0313	0.0320	0.0004	0.0044	0.0040	0.0054	0.0051	0.0050

* Three sets of parameters of beta distribution define three modes of weight function
